# Copper Compounds: A Narrative and Regulatory Review of Agricultural Uses, Risks, and Environmental Assessment

**DOI:** 10.3390/toxics14070632

**Published:** 2026-07-20

**Authors:** Alberto Angioni, Mattia Casula, Virgilio Stillittano, Francesco Corrias

**Affiliations:** 1 Department of Life and Environmental Science, University of Cagliari, 09100 Cagliari, Italy; mattia.casula@unica.it (M.C.); francesco.corrias@unica.it (F.C.); 2 Experimental Zoo Prophylactic Institute of the Lazio and Tuscany Regions, 00178 Rome, Italy; v.stillittano-esterno@sanita.it

**Keywords:** plant protection products, environmental risk assessment soil accumulation, human health, dietary exposure, EFSA

## Abstract

Background: Copper is a trace element involved in key physiological and biochemical processes in humans, animals, and plants, and its homeostasis is tightly regulated. However, excessive exposure may cause adverse health and environmental effects. Copper-based plant protection products contribute to dietary exposure to copper and remain indispensable for the control of several crop diseases. Methods: This review summarizes the current state scientific and regulatory evidence on agricultural copper, identifies major knowledge gaps, and discusses strategies to support its sustainable use. A structured literature review was conducted using the PECO framework to define eligibility criteria, while key principles of the PRISMA 2020 statement were applied to improve the transparency of study identification and selection. Results: The available evidence confirms the dual role of copper as an essential micronutrient and a potentially toxic element, with adverse effects depending on exposure level, chemical form, bioavailability, and physiological status. Although copper remains an effective agricultural tool, long-term use may promote soil accumulation and environmental impacts. Current environmental risk assessment frameworks, originally developed for organic chemicals, do not fully account for the environmental behavior of inorganic metals. The review also examines copper occurrence, biological functions, agricultural uses, environmental fate, toxicity, and regulatory frameworks. Conclusions: Sustainable copper use requires balancing crop protection benefits with human health and environmental protection. Dietary exposure from authorized copper-based PPPs is generally considered negligible according to current EFSA PRIMo assessments, whereas occupational exposure and environmental accumulation remain important concerns. Future environmental risk assessment frameworks should better account for the environmental fate, natural background concentrations, bioavailability, and speciation of inorganic metals such as copper, thereby improving the scientific basis of regulatory decision-making.

## 1. Introduction

Copper is an essential micronutrient for plant and animal health [1,2]. The richest dietary sources of copper include shellfish, seeds and nuts, whole-grain products, wheat bran cereals, organ meats such as liver, chocolate, and some fruits and vegetables [3].

Drinking water conveyed through copper pipes can also contribute to dietary intake [4]. It acts as a cofactor for crucial enzymes involved in photosynthesis, respiration, iron metabolism, and immune function [5]. However, copper can become toxic at high levels [2].

This requires careful management of soil and feeding [5]. Although copper compounds are important for plant and animal nutrition, their main agricultural use is crop protection [6].

Copper compounds are primarily used as plant protection products (PPPs) in agriculture and exhibit broad-spectrum activity against bacteria, oomycetes, ascomycetes, and basidiomycetes, thereby preventing fungal spore germination [7]. In rice, it is used to control tadpole shrimp and algae. Copper has a multisite mechanism of action that minimizes the risk of development of resistant pathogen strains [8].

Copper is applied using air-blast ground-boom sprayers, mechanical dusters, hand-wand sprayers, push spreaders, and automatic metering equipment. Aerial application is rarely used, except in rice cultivation. In addition, copper is one of the few pest control agents certified for use in organic agricultural production and is a critical control tool in this system [9].

Copper does not act as a molecular compound, but as a copper ion (Cu^2+^) released from the formulation after application. Copper ions (Cu^+^ and Cu^2+^), in concentration, interact with nucleic acids, interfere with energy transport, disrupt enzyme activity, and affect the integrity of pathogens’ cell membranes [10]. Both ions have fungicidal and bactericidal activity. Following absorption by fungi or bacteria, copper ions bind to various chemical groups (imidazole, phosphate, sulfhydryl, and hydroxyl) present in many proteins, thereby disrupting their functions. Copper ions can kill pathogenic cells on plant surfaces, but once a pathogen enters host plant tissue, it is no longer susceptible to copper treatments at the prescribed concentrations. Therefore, copper ions exhibit reduced post-infection activity, and higher concentrations can harm the host plant [11].

In aquaculture, it is used to control algal growth in water and several external parasites and pathogens affecting farmed fish [12,13]. Copper exerts species-specific effects in algae, including inhibition of cell division, photosynthesis, adenosine triphosphate (ATP) production, and respiration [14]. Once released into aquatic systems, copper interacts with cell surfaces or is taken up by target organisms, disrupting biological processes and ultimately leading to pest death. In terrestrial agriculture, it acts as a contact pesticide, forming a protective barrier on leaf and plant surfaces, inhibiting fungal spore germination, disrupting membranes, and leading to bacterial cell lysis [15].

Commercial copper products differ in the form of copper, the salts and complexes used, the amount of formulated copper compound in the product, and the amount of metallic copper (active ingredient) [15]. In Europe, the authorized copper forms are copper hydroxide, copper oxychloride, cuprous oxide, copper sulfate, and Bordeaux mixture [15]. The U.S. Environmental Protection Agency (EPA) reported that 11 copper compounds are registered for use in terrestrial and aquatic agriculture, with approximately 5.7 million total acres treated annually and 8.6 million pounds (lbs.) of metallic copper (MeCu) [16]. There are two broad categories of copper-based products: water-soluble, such as copper sulfate, and water-insoluble, such as oxychlorides and hydroxides [15]. Copper-based PPPs include aqueous solutions, wettable powders, dust, flowables, water-dispersible granules, and emulsifiable concentrates. Water-soluble formulations are short-lived, whereas insoluble products release active copper ions over time and are comparatively stable [17]. Dry flowable formulations (concentrated water-dispersible granules with additives that ensure effective dispersion) appear to be the most advanced and user-friendly formulations. Copper compounds are also authorized as micronutrients and fertilizers and are used in feed additives [18]. Copper compounds can be compared by the amount of metallic copper present per liter or kilogram of product [15].

## 2. History

The history of metallic pesticides dates back to 1807, when copper was first recorded as being used as a fungicide to control stinking smut (bunt) in cereals and to reduce sheep foot rot, and later as a wood preservative [19]. In 1845, Morren reported that a mixture of lime, sea salt, and copper sulfate could prevent late blight (*Phytophthora infestans*) on potatoes, but his findings were ignored despite the devastating consequences of the Irish potato famine [20,21]. Similar observations reported in the United States were also dismissed [22]. In 1885, the French scientist Millardet developed the Bordeaux mixture (copper sulfate, hydrated lime, and water), demonstrating the broad-spectrum fungicidal properties of copper. The discovery came from observing that grapevines treated with a green mixture containing copper acetate (verdigris), used on the roadside, resulted in disease-free plants [22].

Beginning in 1889, the Vermont Agricultural Experiment Station applied Bordeaux mixture to all its regular potato fields and reported that late blight could be effectively controlled with this fungicide.

The first U.S. authorisation for a copper-based pesticide was granted in 1956 [23]. Subsequently, EPA Registration Standards and Data Call-In (DCI) required supporting data for reregistration [23].

In Europe, copper compounds were approved on December 1, 2009, following their inclusion in Annex 1 of Council Directive 91/414/EEC of 15 July 1991, through Directive No. 2009/37/EC [24,25].

## 3. Copper in Soil

Only a fraction of the total copper present in soil is bioavailable for plants. This fraction can be estimated using mild chemical extraction techniques based on salts or chelating agents, such as CaCl_2_, DTPA, or EDTA [26]. There is currently no mandatory regulatory approach for determining bioavailable copper; assessment is based on an integrated approach (Table S1) [15,26].

In neutral or slightly acidic soils, copper is concentrated in the upper layer; in the rhizosphere (the interface between roots and soil microorganisms), copper is complexed with organic compounds that regulate plant tolerance mechanisms. Copper tolerance in plants is mediated by phenolic compounds and carboxylic acids, which play a fundamental role in both internal and external strategies [27].

Within the 2002–2009 French Soil Quality Monitoring Network, copper concentrations were accurately mapped. Soil types strongly influenced copper concentrations, ranging from less than 1 mg kg^−1^ in sandy soil to more than 100 mg kg^−1^ in other soil types. However, the survey could not distinguish between natural and anthropogenic sources of copper. Soils with high levels of iron (hydr)oxides or humic substances, such as humic and fulvic acids, may have the greatest capacity to retain copper [28].

Acidic soils, eroded or dug-up soil, are more likely to transfer copper into water systems. Among different soil types, sandy soils have greater transfer than clay soils [29]. After application to field crops, residual copper typically accumulates in the upper 15 cm of soil, bound to organic matter and the fine clay fraction.

A survey of soil samples from 28 European countries indicates that Southern European Regions and some parts of the United Kingdom had the highest total Cu levels, which were strongly influenced by local conditions [30].

The EuroGeoSurveys GEMAS and FOREGS reported the natural geochemical background, the impact of human activities, soil health, and environmental risk assessment, with Cu values of 20.0 mg kg^−1^ in agricultural and 19.3 mg kg^−1^ in grassland soils, and 16.4 mg kg^−1^ for risk assessment. In Europe, average ambient background Cu concentrations in soil range from 11.4 to 17.0 mg Cu kg^−1^ [31,32].

Among crops, vineyard and orchard soils have shown the highest levels of copper contamination, averaging about 49 mg kg^−1^ across the EU, with France showing the highest average concentration (91 mg kg^−1^) [33]. However, several investigations suggest that frequent use of copper-based fungicides is the principal source of contamination in vineyard soils, with levels often exceeding 200 mg kg^−1^ and sometimes reaching 1000 mg kg^−1^ [27]. The Cu concentration increases with vineyard age and is much higher on terraces than on plateaus. EU countries account for 60% of global wine production, with Italy, France, and Spain among the top producers. Therefore, viticulture is a particularly important agricultural sector in the Mediterranean region [34].

The PESCAR project carried out in Croatia showed higher copper concentrations in soils sampled after the growing season across all crop types investigated (olive, stone fruit, grapes, and potatoes), with statistically significant increases in soils under stone fruit plantations and an influence of fertilizer use [35].

High Cu concentrations can severely degrade the soil ecosystem, altering the entire soil food web. Soil organisms support almost every ecosystem function, regulating soil fertility, carbon sequestration, and other processes that affect environmental and human safety. Earthworms are extremely sensitive and tend to accumulate copper in their tissues, so they avoid soils rich in copper. In addition, the soil bacterial communities can be affected by elevated Cu concentration. Consequently, limiting the use of Cu-based products and developing remediation plans to reduce contamination risk and restore appropriate Cu levels may benefit soil organisms and, in turn, their functions [36].

At the European Union level, there is no agreement on copper threshold values for risk assessment. The Copper Voluntary Risk Assessment (VRA) has been used as a scientific reference to define copper levels [37]. Following this assessment, threshold values have been set for soil at 79 mg kg^−1^ DW, drinking water (4.0 mg L^−1^), freshwater, and marine waters, at 7.8 and 2.6 µg Cu L^−1^.

Regarding sediments, the levels are 87, 144, and 338 mg Cu kg^−1^ DW for freshwater, estuarine, and marine sediments, respectively. Finnish and Swedish legislation on soil contamination has proposed guideline and threshold values for Cu in soil of 150 mg kg^−1^ and 100 mg kg^−1^, respectively [38]. The copper threshold of 100 mg kg^−1^ has also been proposed by numerous scientific studies as indicative of unpolluted soils; these values identify a value beyond which further assessment is needed in the area (threshold), and a value denoting an ecological or health risk [39,40]. The Council Directive 86/278/EEC established limit values for copper in soil of 50–140 mg kg^−1^ DW [41]; overall, the natural abundance of copper in the Earth’s crust is around 60 mg kg^−1^ [42]. It should be noted that these values originate from different regulatory or scientific frameworks and therefore should not be interpreted as directly comparable regulatory thresholds.

## 4. Copper and Plants

Copper is one of the 17 essential elements required for the growth and development of cyanobacteria, algae, and higher plants [43]. Healthy plant development requires 5–30 mg Cu kg^−1^ DW, but requirements may vary by crop or even by cultivar [44,45,46]. It participates in various biochemical functions of the plant life cycle, including photosynthesis, respiration, ethylene sensing, ROS clearance, cell wall metabolism, hormone signalling, and regulation of key N-assimilation enzymes [47]. In green plants, epidermal root cells absorb copper ions from the soil matrix, which are then transported through the parenchyma and endodermis, ultimately entering the xylem. Approximately 30% of Cu is stored in chloroplasts, with about 60% localized in the thylakoids, where plastocyanin, a small, soluble copper-containing protein, acts as an electron carrier in Photosystem I (PSI) [48,49].

Cu acts as a structural or catalytic cofactor in essential cellular enzymes that exploit its redox potential, typically present as Cu^+^ or Cu^2+^ [43]. The main biochemical pathways are those involved in the synthesis of proteins, carbohydrates, and chlorophyll.

It is essential for the proper functioning of the electron transport chain in chloroplasts and, through superoxide dismutase, protects chloroplasts against oxidative damage [46]. In addition, copper is involved in lignin formation in cell walls, proper pollen tube growth, and pollen tube viability. The research also highlighted that copper affects the flavour, storage ability, and sugar content of fruit [50].

### Copper Deficiency in Plants

Copper is evenly distributed in the plant but is relatively immobile in the phloem. Therefore, a constant supply is required throughout the growing season, and precise homeostasis is necessary [51,52].

Critical Cu deficiency generally ranges between 0.001 and 0.005 mg g^−1^ DW. In plants, Cu deficiency typically affects gene expression, the regulation of various biological processes, and root and leaf morphology, ultimately leading to growth retardation, increased senescence, and reduced productivity [44]. Photosynthesis is the primary target of Cu deficiency. The typical symptoms first appear in young leaves, manifesting as chlorosis at the leaf tips, leaf-edge curling, leaf deformity, or even necrosis (death) [43]. The death of the growing points often leads to excessive tillering in cereal crops and excessive branching in dicots (non-grass crops) [53]. Some vegetables show a blue-green colour before advancing to chlorosis. Decreased fertility in Cu-deficient crops results from multiple factors, including stunted stigmas, reduced pollen fertility, and reduced pollination capacity. Reduced seed and fruit yields are mainly due to male sterility [54].

Copper deficiency in crops can be addressed through several methods, including soil application, foliar sprays, and fertigation. The application of a copper-containing spray allows a more rapid uptake by the plant than from the soil. Foliar applications can be particularly effective for addressing acute deficiencies. Soil fertilization should use dry granular NPK fertilizers containing copper to ensure uniform distribution across the field. In a fertigation system, copper can be supplied to plants through soluble fertilizers that readily dissolve in water [55].

Early interventions can prevent more severe problems. When implementing a fertilizer program, the first step should be soil testing to determine nutrient needs, along with plant analyses. A record must be kept of the total amounts applied to fields to avoid residual effects.

## 5. Copper in Aquaculture

The macro- and trace-mineral requirements of many farmed aquatic species have been established, and maintaining appropriate copper (Cu) levels is essential for growth and well-being. However, overdosing on water-borne minerals can also be toxic to aquatic animals. Scientific research has been conducted to determine the optimal mineral requirements for different fish species and sizes, as well as for different feed types and farming conditions [12,13,56,57].

The copper forms used in aquafeed preparation mainly consist of inorganic forms (oxide, chloride, carbonate, sulfate); among these, CuSO_4_·5H_2_O is the most commonly added to aquafeed [12,13,57].

However, the bioavailability of inorganic copper can be influenced by dietary interactions and physiological factors. In contrast, organic copper forms complexes with amino acids, peptides, and other organic compounds that are less susceptible to antagonistic interactions during intestinal absorption [12].

The use of copper compounds in aquaculture is not limited to nutritional purposes; it also includes direct use as an algaecide and herbicide in water, molluscicide applications, snail and mussel control, sewer root control, and pathogen control on ornamental trees [13,58,59]. As an algaecide/herbicide, copper interferes with photosynthetic processes, although copper compounds have not been classified into a specific herbicide mode-of-action group by the Weed Science Society of America (WSSA).

## 6. Copper and Human Health

Copper is an essential element in humans and animals; it acts as a cofactor in several metalloenzymes involved in physiological and biochemical processes (e.g., cytochrome C oxidase and Cu/Zn superoxide dismutase) [60,61].

The adult human body is estimated to contain 1.4–2.1 mg of Cu kg^−1^ body weight (bw), corresponding to approximately 50–150 mg in total. Although approximately 40% of total body copper is present in skeletal muscle because of its large mass, the liver is the principal organ responsible for copper storage and homeostasis [60].

In 2015, the European Food Safety Authority (EFSA) established the Dietary Reference Values (DRVs) for copper [61]. However, defining more precise reference values, such as Average Requirements (ARs) and Population Reference Intakes (PRIs), was not possible due to several limitations. These include the lack of reliable biomarkers for assessing copper nutritional status and the limited robustness of studies on copper metabolism. Consequently, Adequate Intakes (AIs) were established in place of the previous values (Table 1). These represent intake levels considered sufficient to meet physiological requirements and were derived from observed dietary intakes in European populations, with limited clinical evidence supporting them [62].

In the liver, copper is sequestered by metallothionein (MT), a key protein involved in the homeostasis of both copper and zinc. Nevertheless, there remains a lack of reliable human data on the effects of long-term exposure to low levels of copper, a critical gap in current knowledge.

Copper’s importance in biological systems stems largely from its ability to cycle between the Cu(I) and Cu(II) oxidation states, thereby reversibly accepting and donating electrons in essential redox reactions and antioxidant defense [63,64,65,66]. Copper undergoes complex homeostatic processes that ensure a constant and sufficient supply of the micronutrient while avoiding excessive free metal levels in the cell, thereby preventing the consequences of Cu overload and Cu deficiency. Homeostasis is controlled by transcriptional and selective transport mechanisms mediated by cysteine-, methionine-, and histidine-rich proteins [31,63]. The duodenum and the small intestine absorb 20–60% of dietary copper; the remainder is released into the bloodstream, bound to soluble chaperones such as albumin, transcuprein, histidines, and macroglobulins, transported to the liver, and then distributed or stored. Excess is eliminated in feces or via biliary excretion [67]. Copper from soluble salts is absorbed more readily than from oxides [68]. Many components, such as phytate, fiber, vitamin C, and some sugars, reduce copper bioavailability, whereas high protein intake increases it [63].

### Copper Deficiency in Humans

Adequate intakes vary according to age and sex. In women, it also changes during pregnancy and lactation, increasing by 0.2 mg day^−1^ relative to non-pregnant levels (1.3 mg day^−1^) to cover fetal tissue deposition and copper secreted in breast milk [61]. Serious deficiencies in humans are rare. However, the population at risk of deficiency is broad, including newborns with low birth weight and those with inadequate dietary intake due to single-nutrient feeding, owing to Cu intake below 0.1 mg Cu kg^−1^ bw day^−1^ [27]. Clinical conditions such as diabetes and alcoholism, vegetarians, or other restrictive diets can lead to inadequate absorption of copper in humans. Limited absorption can be attributed to competition or sequestration by other molecules, or to the inability to incorporate into cuproenzymes. A notable genetic cause of copper deficiency is Menkes syndrome, a rare disorder resulting from mutations in copper-transporting ATPases and leading to severe copper deficiency [63]. Copper deficiency can cause anemia, neutropenia, and bone marrow dysplasia, which may be under-recognized; numerous case reports have been published [69,70,71].

## 7. Copper Pesticides

Although some copper compounds have occasionally been described as systemic, the evidence supporting this characteristic is lacking. Copper compounds are considered non-systemic, and the amount of copper in plant tissue remains too low to exert a toxic effect on microorganisms. No specific studies have investigated the metabolism and distribution of copper residues in plants following foliar application [15].

Technical purity varies by compound (hydroxide, oxychloride, Bordeaux mixture, tribasic sulfate, or oxide) (Table 2). No FAO specification for basic copper sulfate has been reported [72]. Impurities in copper-based products are subject to strict limits, expressed on a total copper basis: arsenic and cadmium must not exceed 0.1 mg g^−1^ of total Cu, lead 0.3 mg g^−1^, and nickel 1 mg g^−1^ [15]. Moreover, the maximum limits for other impurities in the technical material have been set: cobalt at 3 mg kg^−1^, mercury at 5 mg kg^−1^, chromium at 100 mg kg^−1^, and antimony at 7 mg kg^−1^ [15]. The efficacy of copper compounds can be optimized by reducing particle size (micronization) or through microencapsulation, allowing for lower modern dosages [1,6,73].

Copper is a relatively nonspecific bactericide and fungicide and can kill naturally occurring microorganisms on leaves as well as those applied as biocontrol agents, including *Bacillus* sp., *Trichoderma*, and others. Therefore, it remains essential for controlling numerous fungal and bacterial diseases in various crops (Table 3). However, several bacterial pathogens have developed resistance to certain copper ion concentrations (e.g., *Pseudomonas syringae, Erwinia amylovora, and Xanthomonas campestris* pv. vesicatoria), and copper is not effective against some pests (e.g., *Sclerotinia blight*, some *Phytophthora* species, and *Rhizoctonia*) [75,76,77].

Once copper enters microbial cells, it can bind to several protein functional groups (imidazoles, phosphates, sulfhydryls, hydroxyls), thereby disrupting protein and enzyme function and causing cellular and membrane damage [28,78].

In mollusks, it disrupts the function of the surface epithelia and peroxidase enzymes.

### Regulatory Framework and Restrictions

The regulatory framework for copper compounds varies worldwide, leading to different approaches for agricultural applications and dietary limits (Table S2). Overall, international regulatory frameworks adopt similar health-based drinking-water values (generally 2 mg/L) and maintain authorization systems for copper-based pesticides. The European Union applies a more precautionary regulatory approach by restricting cumulative agricultural copper inputs and classifying copper compounds as Candidates for Substitution [73]. In contrast, no internationally harmonized soil quality thresholds exist, and environmental risk assessments are based on assessments during the PPP revision rather than on fixed concentration limits (Table S2).

Copper PPPs are registered in almost all European countries; however, they are not registered as active substances in five of the 27 EU member states (Denmark, Sweden, Finland, the Netherlands, and Estonia) [9].

Copper compounds are reported in Regulation (EC) No. 1107/2009 of the European Parliament and of the Council of 21 October 2009, concerning the placing of plant protection products on the market [79]. Later, they were included in the Annex to Commission Implementing Regulation (EU) No. 540/2011 of 25 May 2011 [74]. Copper has been approved as an active substance subject to substitution, in accordance with Article 24 of Regulation (EC) No. 1107/2009 [79,80]. Approval for copper compounds was renewed by EU Regulation 2018/1981, valid for 7 years from 1 December 2019 to 31 December 2025 [81]. A position paper was prepared by the European Union Copper Task Force (EUCuTF) in 2023, requesting the removal of copper compounds from Candidate for Substitution status under (EC) 1107/2009, in accordance with the new CLP revision, which states that the PBT (persistent/bioaccumulative/toxic) and vPvB (very Persistent and very Bioaccumulative) status shall apply to all organic substances, including organometals [82].

The Commission Implementing Regulation (EU) 2025/1489, in Part E of the Annex to Implementing Regulation (EU) No. 540/2011, has extended the approval date to “30 June 2029”. This modification is not related to intrinsic copper issues but to bureaucratic delays [83].

The maximum application limit is 28 kg of metallic copper per hectare over 7 years (average of 4 kg ha^−1^ year^−1^), regardless of use. All products must undergo a comparative assessment to examine possible alternatives [81].

The conditions for using copper are the same in organic farming and conventional farming [84].

A roadmap was presented in France in 2019 to reduce the use of copper in agriculture, following significant variability in its use across regions (from 1.6 kg Cu ha^−1^ year^−1^ in Alsace to more than 6 kg Cu ha^−1^ year^−1^ in Champagne and Languedoc-Roussillon) and crops [33].

## 8. Copper in Organic Agriculture

According to the Research Institute of Organic Agriculture (FiBL), 1.96 × 10^5^ km^2^ of Europe is dedicated to organic farming, accounting for around 11% of the total European farmland, with a target of 25% by 2030 under the Farm-to-Fork Strategy [18,85]. Moreover, the organic market is increasing constantly, making Europe the second-largest organic market in the world [86].

Copper is explicitly permitted in organic farming as a plant-protection product and as a fertilizer under EU Regulation 2021/1165 [85]. Copper is essential for plant protection in organic farming due to its broad-spectrum activity against bacteria and fungi and its low cost; moreover, it has only a few less effective alternatives. Organic farming depends almost exclusively on its use [87]. Available alternatives include agronomic measures (e.g., crop rotation, canopy management, resistant varieties), biological products (e.g., *Bacillus* spp.), and other permitted products (e.g., sulfur, which is only active against fungi; lime; and essential oils) [88,89].

Various surveys across several European countries show that use varies considerably by crops and region [90,91]. Moreover, a survey conducted across 12 European countries among organic farmers found that about 53% of the maximum authorized amount was used [9].

In the UK, the Soil Association permits its use on certified organic crops only in cases of a major threat of selected compounds, such as copper sulfate, copper hydroxide, cuprous oxide, copper oxychloride, copper ammonium carbonate (at a maximum concentration of 25 g L^−1^), and copper octanoate, and the maximum amount is 6 kg ha^−1^ year^−1^.

A survey in Switzerland found that organic farmers use, on average, between 3 (cabbage and cucumber) and 80% (pear) of the maximum permitted amount of copper, depending on the crop [92].

In fertilizers containing macronutrients Nitrogen (N), phosphorus (P), potassium (K), magnesium (Mg), and calcium (Ca), copper is considered a contaminant, and maximum levels are defined [93]. In organic fertilizer, the concentration of copper must not exceed 300 mg kg^−1^ dry matter by Regulation (EU) 2019/1009 [94]. Organic soil has been found to contain lower levels of metals, including copper, than conventional farming [42].

The complete elimination of copper would currently result in significant yield losses in many crops. Decision-makers, producers, and organisations in organic farming management are still dealing with whether to limit copper or abandon it altogether. This decision also reflects the primary task of risk evaluators and environmental safety agencies (such as EFSA and ANSES). More work should be done to establish the elements of the available alternatives, their efficacy, and the problems or obstacles associated with their adoption.

## 9. Copper Toxicity

Exposure–outcome associations relevant to human health, plants and the environment were investigated through a structured literature review. The PECO (Population, Exposure, Comparator and Outcome) framework was used to formulate the research questions and define the eligibility criteria for the three thematic areas considered (human health, environmental toxicity, and phytotoxicity). The literature search was performed using four sources (EFSA, PubMed, Web of Science, and Scopus). References were managed in Zotero, whereas title/abstract and full-text screening were performed in Rayyan. Identification, screening, and selection of publications followed key reporting principles of the PRISMA 2020 statement to improve the transparency and reproducibility of the literature search process. Given the broad scientific and regulatory scope of the present work, the review was designed as a structured narrative review rather than a formal systematic review; consequently, PRISMA was used only to guide the reporting of the literature search and study selection, and no protocol registration, risk-of-bias assessment or quantitative evidence synthesis was undertaken. Potentially relevant articles were evaluated in full text against the PECO scenarios, with inclusion and exclusion criteria defined (Table 4 and Table 5).

### 9.1. Human Toxicity

The structured literature search guided by the PECO framework identified the studies included in the overview of human toxicity (Table 6, Tables S3 and S4).

Human evidence: cases of copper poisoning in humans related to copper ingestion date back to the 1900s. They were related to the unsafe use of copper cooking devices, outdated water delivery systems, and the absence of DPI during PPP preparation or field distribution [73].

Excess copper can induce oxidative stress by generating ROS, which damage lipids (peroxidation), proteins, and DNA [78,95]. Copper ions in the intracellular environment are more active than Fe^2+^ ions in catalyzing the Fenton reaction in the human body, producing highly reactive hydroxyl radicals from hydrogen peroxide [96]. Copper deficiency is also harmful, leading to reduced antioxidant defenses, anemia, pancytopenia, and ataxia [70]. Copper’s adverse effects (particularly cytotoxicity) can vary in severity. Nausea, vomiting, and abdominal pain following acute ingestion, with transient effects at low doses but recurring with high chronic exposure, have led some authors to suggest a reference dose (RfD) of 0.04 mg Cu kg^−1^ day^−1^ for oral exposure in adults and children [97]. Copper compounds may cause skin sensitization in susceptible individuals, and eye exposure can result in conjunctivitis, inflammation of the eyelid lining, corneal damage, and opacity [98].

Menkes syndrome and Wilson disease are rare genetic disorders of copper metabolism caused by mutations in copper-transporting ATPases: Menkes causes severe copper deficiency, while Wilson disease causes toxic copper accumulation [99,100].

Individuals subjected to high chronic exposure to copper compounds showed elevated levels of aspartate transaminase (AST) and alanine aminotransferase (ALT), as well as rare cases of copper accumulation-related liver disease. Systemic copper accumulation showed the liver as the primary target organ, with accumulation also occurring in blood and secondary tissues, with a correlation between exposure and serum copper levels [60,101,102,103,104,105,106].

Experimental animal toxicology: Experimental studies have been fundamental for characterizing copper toxicity and establishing dose–response relationships. Copper hydroxide has less acute toxicity than copper sulfate, with an oral LD50 in rats of 833 mg Cu kg^−1^. The relevant short-term NOAEL used in regulatory human health risk assessment is 16 mg Cu kg^−1^ bw day^−1^, derived from a 90-day rat study, replacing the previous regulatory value of 10 mg Cu kg^−1^ bw day^−1^ [60,107]. In contrast a no-observed-adverse-effect concentration (NOAEC) of 0.2 mg Cu m^−3^ was established for local inhalation effects [60]. Additional toxicology studies demonstrated dermal adsorption is limited, with acute dermal LD50 values exceeding 5000 mg Cu kg^−1^ bw in rats [52].

Regulatory human health assessment: regulatory reference values have been established by integrating human observations with experimental animal data using a weight-of-evidence approach. EFSA Scientific Committee established an Acceptable Daily Intake (ADI) at 0.07 mg Cu kg^−1^ day^−1^ bw based on human infant data and, in accordance with WHO values, equivalent to 5 mg Cu day^−1^ for adults [60,62].

Copper variants are not classified or proposed to be classified as toxic for reproduction category 2 or carcinogenic category 2, in accordance with the provisions of Regulation (EC) No 1272/2008 [108], and therefore, the conditions of the interim provisions of Annex II, point 3.6.5 of Regulation (EC) No 1107/2009 concerning human health for the consideration of endocrine disrupting properties are not met [79].

Regarding PPP, a data gap was evident for stabilizers, as no toxicological information has been provided for these chemicals. Toxicological studies on copper sulfate could be used to assess the risk of the five variants [15].

Despite toxicological evidence, no critical toxicological endpoints for human health were identified, mostly because copper is a naturally occurring element and is regulated by the body. Therefore, the US EPA has not implemented any risk mitigation measures to address human health concerns [16].

Overall, the regulatory risk assessment did not identify unacceptable dietary, residential, occupational, or aggregate risks under the authorised conditions of use. However, specific operator and re-entry worker exposure scenarios may exceed the AOEL under certain crop or application conditions, requiring appropriate risk mitigation measures, such as PPE or use restrictions [109].

### 9.2. Phytotoxicity

The structured literature search guided by the PECO framework identified the studies included in the overview of plant toxicity (Table 7).

Toxic Cu concentrations vary significantly among plant species and genotypes, leading to substantial differences in Cu tolerance between species [110,111,112]. It is very toxic to algae at high concentrations [113]. For example, stone fruit leaves are more sensitive to copper phytotoxicity than apple leaves. Copper-tolerant plant families include Cruciferae, Caryophyllaceae, Gramineae, Leguminosae, and Asteraceae [6].

Copper increases cell membrane permeability and the leakage of cellular constituents. However, the most important effect of copper on plants and algae is its inhibition of chlorophyll biosynthesis and photosynthesis, through structural damage to thylakoids, alterations in their lipid composition, lipid and protein oxidation, inhibition of photophosphorylation, and reduced efficiency of ribulose-1,5-bisphosphate carboxylase/oxygenase [114,115,116,117,118].

Cu phytotoxicity is evident at the root level, with a clear decrease in root elongation, abnormal root branching, thickening, and darkening. Because of this altered root development, the root’s ability to absorb water and other nutrients is compromised. Moreover, Cu can influence root P uptake, highlighting interactions among different nutrient uptake mechanisms at concentrations of 100–400 mg kg^−1^ DW [27]. Cu toxicity is closely related to Fe deficiency; many Cu proteins have functional counterparts that use Fe as a cofactor, and growth on a Cu-rich substrate is commonly associated with decreased Fe content in roots and leaves [119]. Consequently, plant phenotypes associated with Cu toxicity resemble those associated with Fe deficiency, including leaf chlorosis, reduced leaf chlorophyll content, and increased oxidative stress [51].

Among plant physiological processes, seed germination represents an important stage, being the start of a new plant. This phase is highly susceptible to heavy-metal contamination, and even low Cu concentrations can reduce seed germination rates, thereby inhibiting bud growth and root formation [120].

Moreover, prolonged dry conditions, the presence of surfactants in plant protection products, and acid-forming products may increase plant foliage injury by releasing higher levels of copper ions [121,122,123,124].

The production of ROS, together with the activation of the antioxidant defense system and increased levels of ROS-modified lipids and proteins, has been associated with copper-induced inhibition of photosynthesis and inhibition of PSII acceptor on its oxidizing side, namely the quinone acceptor QA, pheophytin-QA-Fe, region, non-heme type Fe^2+^, and secondary quinone acceptor QB [125,126].

A reduced electron flow, associated with metal-ion interference with the photosynthetic apparatus, was proposed based on data showing modifications in electron donation from Tyrz to P680 and in its microenvironment [127]. Additionally, the photosystem can be deactivated by replacing the central magnesium atom of chlorophyll with metals such as mercury, copper, or cadmium [117]. Nevertheless, the precise location of the copper-inhibitor binding site remains unknown.

Some evidence suggests that exposure to high concentrations of Cu reduces plant uptake of macronutrients, such as calcium (Ca), potassium (K), magnesium (Mg), and phosphorus (P), and trace elements, such as Fe, Mn, or Zn, or alters the distribution of these elements in roots and shoots [126,128].

### 9.3. Copper in the Environment and Related Toxicity

The structured literature search guided by the PECO framework identified the studies included in the overview of environmental toxicity, considering its fate in soil and aquatic systems (Table 8).

The main environmental concern relates to the long-term accumulation of copper in agricultural soils.

Copper generally shows limited vertical mobility, except under specific drainage conditions or in very acidic environments, where the risk of groundwater contamination increases [129,130,131]. The primary source of copper emissions into soil is agriculture (over 70%) [92].

Copper in aquatic systems is rapidly bound to minerals and precipitated as insoluble inorganic salts or bound to organic matter. It was estimated that dissipation in the aqueous phase could range from 4 to 30.5 days [15].

The main gaps highlighted in the EFSA peer review were a high risk to aquatic organisms (fish, invertebrates, and aquatic plants) and issues related to soil macroorganisms [15].

Copper transfer from treated fields to adjacent rivers increases during larger rainfall events and during soil erosion, reaching a few to tens of μg L^−1^ in surface waters and tens to hundreds of mg kg^−1^ in sediments in French freshwater ecosystems [132,133,134].

Copper concentrations in aquatic ecosystems may originate from natural sources but can also result from anthropogenic sources, e.g., agricultural fungicide and herbicide runoff, and depend on several water quality parameters, including pH, alkalinity, and dissolved organic carbon. Anti-fouling paints used to control biofouling on ships can contribute significantly to contamination of marine waters, sediments, and biota [135].

Among different uses, copper concentrations in the rhizomes of *Posidonia oceanica* varied along the plant [136,137]. Shellfish and plankton contamination showed levels averaging 353 μg g^−1^ DW and 59 μg g^−1^ DW, respectively, in the digestive gland and in 6–60 μm-sized samples [33].

The high toxicity of copper in fish and aquatic invertebrates has been attributed to its rapid binding to gill membranes, which inhibit essential sodium and potassium transport enzymes, thereby disrupting osmoregulatory processes [138,139].

For all representative uses, it was concluded that there is an elevated risk to fish, algae, and aquatic invertebrates when used on grapes, but not on tomatoes and cucurbits, in the field (if mitigation measures are considered) and in the greenhouse [15].

Bordeaux mixture and Copper (I) oxide had a negligible risk for non-target arthropods; on the contrary, copper hydroxide, copper oxychloride, and tribasic copper sulfate showed a considerable risk.

For earthworms and other soil macroorganisms, a substantial risk was concluded at Tier 1. Studies in vineyards suggest that lumbricids adapt, allowing them to tolerate higher concentrations [140].

## 10. Risk Assessment and Environmental Fate for Regulatory Purposes

### 10.1. Dietary Risk Assessment

The purpose of the EFSA scientific opinions published in 2022 and 2023 was to assess the safety of copper use in relation to human health [60,62]. In the most recent framework in 2026, EFSA outlined the methodology to be used by applicants and Member States for conducting consumer risk assessments of PPPs containing copper compounds across different regulatory frameworks [141].

New exposure assessments are not required for already authorized use of copper compounds unless modifications to existing MRLs are proposed. Only when dealing with a representative or intended use that requires a modification to the existing MRL should new exposure calculations be performed [107].

Regarding safety thresholds, the Scientific Committee on Food (SCF) established in 2003, and EFSA confirmed in 2006, a tolerable Upper Intake Level (UL) of 5 mg day^−1^ for adults, applying an uncertainty factor (UF) of 2 to account for inter-individual variability [142,143]. In 2008, during the EFSA peer-review process, a reference point (RP) of 0.15 mg kg^−1^ bw day^−1^ (approximately 10 mg day^−1^ for a 70 kg adult) was identified, consistent with the Acceptable Daily Intake (ADI) [144].

This value was derived primarily from animal studies (90-day rat study with a NOAEL of 16 mg kg^−1^ bw day^−1^) and supported by data from the World Health Organization (WHO, 1996) and long-term dog studies (NOAEL of 15 mg kg^−1^ bw day^−1^) [107,144]. This reference point was subsequently confirmed by EFSA in 2018 [15].

In 2023, the ADI was revised downward to 0.07 mg kg^−1^ bw day^−1^ and confirmed in 2026 [107].

Dietary exposure assessments, conducted using EFSA databases and the Pesticide Residue Intake Model (PRIMo), indicate that the contribution of copper residues resulting from plant protection products (PPPs), fertilizers, and food/feed additives use is small compared to total dietary intake, although, under certain dietary scenarios, chronic exposure to total copper may exceed the ADI for one diet [145]. However, the use of copper in agriculture (fertilizers and plant protection products) can lead to accumulation in soil, posing an environmental rather than a dietary concern [141].

Dietary exposure assessments indicate that, under a specific dietary scenario, chronic exposure to total copper may exceed the ADI for one diet evaluated by EFSA. In addition, infant formulas may be a significant contributor to copper intake during infancy and therefore require specific consideration [62]. Nevertheless, under the currently authorised conditions of use, no major concerns have been identified for the remaining population groups.

### 10.2. Non-Dietary Risk Assessment (Operator, Worker, Resident, and Bystander)

Copper-based plant protection products pose potential risks to agricultural operators and workers re-entering treated fields, in particular, vineyards and tomato fields. Therefore, operators should wear personal protective equipment (PPE) during mixing, loading, and applications to ensure that exposure remains below the Acceptable Operator Exposure Level (AOEL). In addition, in certain scenarios, particularly in vineyards, copper compounds can persist on leaf surfaces, and re-entry workers’ exposure to treated fields may exceed acceptable levels even when PPE is used [15]. It should be noted that these calculations were performed during the regulatory assessment using exposure and risk assessment models that were originally developed for organic substances and may not fully account for the specific environmental behavior of mineral compounds. Such models rely on standardized exposure assumptions and generally use total contaminant concentrations, without explicitly accounting for natural background levels, site-specific geochemical conditions, or metal bioavailability. These limitations are particularly relevant for persistent inorganic contaminants such as copper, for which bioavailability and naturally occurring soil concentrations strongly influence environmental exposure and risk characterization. Consequently, the applicability of generic risk-assessment models to mineral substances remains an area of ongoing scientific and regulatory discussion [146].

The setting of an acute reference dose (ARfD) or an acute acceptable operator exposure level (AAOEL) was not deemed necessary [60].

For the general population (residents and bystanders), exposure is generally below safety thresholds, although under specific conditions, children may exceed AOELs in specific exposure scenarios.

### 10.3. Environmental Fate (Soil)

The exposure assessment in soil begins with consideration of background levels. These data were reported in the RAR of copper compounds and in the EFSA statement on transition metals [15,146]. The overall background-level median value has been set to 11.5 mg Cu kg^−1^ soil, with substantial differences among soil sources (vineyards: 66.4, arable fields: 13.4, and orchards: 48.3 mg Cu kg^−1^ soil) [15]. PEC soil estimation for environmental accumulation assessments should be based on the FOCUS approach, including the background value, and the plateau concentration at 5 and 20 cm for permanent and arable crops, respectively. The use of the FOCUS models for soil exposure showed important limitations, as they were developed for organic substances and do not account for background concentrations and copper bioavailability. Therefore, interpretation of model outputs requires consideration of site-specific soil characteristics.

### 10.4. Environmental Fate (Groundwater)

Groundwater assessment of copper showed values below the legal limit of 2 mg L^−1^ set by Directive (EU) 2020/2184 [147]. Groundwater contamination is generally not expected under the representative scenarios evaluated by EFSA at reduced application rates (4 kg Cu ha^−1^ year^−1^), although local soil properties (e.g., pH, texture, organic matter, and drainage conditions) may influence copper mobility.

### 10.5. Environmental Fate (Surface Water and Sediment)

Based on current knowledge of predictive models, PECsw and PECsed for copper can be derived using the current version of FOCUS STEP 1-2, following the experts’ recommendations during the EU Peer Review [15].

Surface water and sediments monitoring data showed median values of 0.88 μg L^−1^ and 37 mg kg^−1^, respectively. However, EFSA’s conclusions highlighted a critical gap: a lack of data on potential increases in background levels in stream sediments [15]. When performing an assessment, it is scientifically more appropriate to consider the dissolved copper concentration, given that in a realistic water/sediment system, total copper in the water column declines very rapidly due to precipitation/settlement of suspended matter/adsorption onto sediment.

For this reason, different correction factors were recommended: studies submitted for the purpose of renewal proposed a conservative value of 10 (data available only in renewal reports); the RMS, during the renewal evaluation, proposed a value of 2; and the EU Peer Review agreed that no correction factor is needed [15]. Correction factors are left to single member states, for example. According to the Italian Ministry of Health a sufficiently conservative value of 3 could be adopted and applied to the PECsw in Focus STEP 1 and STEP 2 (guidelines available only for dossier reviewers).

Additional monitoring data on dissolved copper levels were provided, and EUCuTF has initiated ongoing trials. No conclusive assessment could be made due to the large variability in copper levels in European water bodies and sediments, from which background concentrations could be derived.

### 10.6. Ecotoxicology Risk Assessment

Regarding ecotoxicology in the peer review, it has been reported: at Tier 1, a high acute and/or long-term risk was identified for birds, mammals, aquatic organisms, and sediment dwellers for the representative uses evaluated. Subsequent refinements led to different conclusions depending on the taxonomic group, endpoint, and representative use, as described in the EFSA peer review [15]. The standard risk assessment scheme for birds and mammals, the Multiple Application Factor (MAF) and the Time-Weighted Average (TWA), cannot be applied to copper. The risk assessment should use a Weight of Evidence (WoE) approach based on literature and monitoring data. The acute risk to birds and mammals does not seem to be a major issue in risk assessment.

The Regulatory Acceptable Concentrations (RAC) for freshwater and sediment-dwelling organisms are below the natural background levels of copper in water and sediment from several unspoiled environments; therefore, to ensure a proper outcome of the risk assessment, application doses in g ha^−1^ year^−1^ should be below those considered efficacious for controlling pests. The Biotic Ligand Model (BLM) could be used to calculate site-specific “safe” RAC thresholds, accounting for toxicity from free copper ions in the media [15]. However, it was not accepted during the EU Peer Review process; nonetheless, Italy considers this approach crucial for conducting a proper aquatic risk assessment and suggests that normalized endpoints could be used in accordance with the VRA for copper [37].

The risk assessment for sediment-dwelling organisms is at a crossroads. The large amount of data generated by different methodologies has not yielded robust, reliable values, often resulting in RAC values below the natural background level in sediments. Therefore, additional data on copper background concentrations in sediment and a more complete and comprehensive toxicity data package are required to support a more scientifically robust risk assessment.

Data on bee toxicity should be provided in accordance with EU Regulations 283/2013 and 284/2013 [148,149].

For risk assessments for which the data are not robust or reliable, such as those involving earthworms, other soil macro-organisms, and non-target organisms, the submitted alternative approaches will be evaluated on a case-by-case basis.

## 11. Conclusions

This narrative and regulatory review highlights the complexity of copper risk assessment, arising from its dual role as an essential micronutrient and a plant-protection active substance. The available evidence indicates that, under the currently authorized uses of copper-based plant protection products and according to EFSA PRIMo dietary exposure assessments, the contribution to dietary exposure is generally negligible for the general population.

Total dietary copper exposure from all sources, including naturally occurring copper in food and drinking water, may exceed health-based guidance values in specific dietary scenarios.

Occupational exposure and long-term environmental accumulation remain the principal regulatory concern.

The literature consistently shows that repeated applications of copper promote accumulation in agricultural soils, particularly in vineyards and orchards, with potential adverse effects on soil organisms and aquatic ecosystems. In contrast, evidence for chronic adverse effects in humans under current dietary exposure scenarios associated with PPP use is limited, although important uncertainties remain regarding long-term low-level exposure and the lack of reliable biomarkers of copper status.

The review also identified considerable heterogeneity in study design, exposure characterization, and toxicity endpoints, limiting direct comparisons among studies and preventing quantitative synthesis of evidence. In addition, regulatory approaches differ substantially among regulatory jurisdictions, particularly regarding environmental threshold values and soil quality criteria.

Current environmental risk assessment methodologies, largely developed for organic chemicals, may not fully account for the persistence, natural background concentration, speciation, and bioavailability of inorganic metals such as copper.

Future regulatory developments should focus on refining exposure and risk assessment methodologies, improving environmental monitoring, harmonizing threshold values, and supporting the development of effective alternatives and sustainable management strategies that preserve crop protection while minimizing environmental impacts.

This review has several limitations. Although the literature search followed a structured PECO-based approach and key PRISMA 2020 reporting principles, no protocol was prospectively registered, no formal risk-of-bias assessment was performed, and no quantitative evidence synthesis or meta-analysis was undertaken. Consequently, the conclusions should be interpreted within the context of a structured narrative review.

## Figures and Tables

**Table 1:** Adequate intakes (AI) for adults, children, and infants.

		AI (mg day^−1^)
Adults	Men	1.6
	Women	1.3
	Lactating women	1.5
	Infants 0–6 months	0.28
	7–11 months	0.4
Children	1–2 years	0.7
	3–9 years	1.0
	10–18 years males	1.3
	10–18 years females	1.1

**Table 2:** Technical and minimum purity of copper compounds [74].

Compound	Range (g kg^−1^)		Minimum Purity
	Min	Max	(g kg^−1^)
Copper hydroxide	583	625	≥573
Copper oxychloride	570	581	≥550
Bordeaux mixture	257	276	≥245
Tribasic copper sulfate	490	543	≥490
Copper oxide	858		≥820

**Table 3:** Major agricultural uses of copper compounds.

Crops	Pest
Vines	downy mildew (*Plasmopara viticola*)
Apple trees	apple scab (*Venturia inaequalis*)
Potatoes	late blight (*Phytophthora infestans*)
Citrus fruit	citrus canker (*Xanthomonas citri*), melanosis (*Diaporthe citri*), brown rot (*Phytophthora* spp.)
Nuts	walnut bacterial blight (*Xanthomonas arboricola* pv. *juglandis*)
Horticultural (onions, pepper, cucurbit)	bacterial soft rot, angular leaf spot, phytophthora, bacterial fruit blotch
Rice	algal mats, tadpole shrimp
non-agricultural applications	algaecide, aquatic herbicide, molluscicide, root control in sewers
aquaculture	aquatic algae and protozoans, antifouling paints

**Table 4:** PECO criteria used to define the research questions and eligibility criteria for the structured literature search.

Scenario	Population	Exposure	Comparator	Outcome
1 Human/animals	Operators, workers, bystanders, residents/rodents, cattle, poultry,	Exposure to copper through drinking water, food, or inhalation of dust or fumes containing copper	Populations not exposed or with lower exposure levels (baseline or regulatory limits WHO/)	Hepatotoxicity (elevated liver enzymes), gastrointestinal disturbances, systemic copper accumulation, and blood biomarkers of copper exposure
2 Soil and aquatic system	Soil and aquatic organisms	Copper contamination in soil and aquatic system (from fertilizers, pesticides), concentration in mg kg^−1^	Uncontaminated soil/water or soil/water with natural background concentrations	Reduced microbial activity, altered biodiversity, toxicity to soil/aquatic fauna, and changes in biogeochemical cycles
3 Plant	Cultivated plants (e.g., lettuce, wheat, grapevines)	Copper in the soil or nutrient solution; root uptake and accumulation in plant tissues	Plants grown in uncontaminated soil or soil with low concentrations of copper	Reduced growth (decreased biomass), leaf chlorosis, oxidative stress, cellular damage, impaired photosynthesis

**Table 5:** Inclusion and exclusion criteria adopted for article selection.

Scenario		Population	Exposure	Comparator	Outcome
1 Human/animals *	Inclusion	Studies on humans and animals	Measured copper exposure (water, diet, air)	Inclusion of a control group or comparison	Health outcomes (liver, gastrointestinal, biomarkers)
	Exclusion	Studies in vitro	Non-specific or multiple exposure without separate data	No comparator or control group	No biological endpoint was evaluated
2 Soil and water	Inclusion	Soil and aquatic organisms	Copper concentration in the soil and water	Control area	Ecotoxicological effects (microorganisms, wildlife)
	Exclusion	Chemistry-only (without biology)	Mixed metals (not separated)	No comparison	No biological endpoint
3 Plant	Inclusion	Cultivated plants	Exposure to Cu (soil or solution)	Control (low Cu)	Growth, physiology, stress
	Exclusion	Non-biological or model studies	Unquantified data	Lack of an appropriate control	Only vague qualitative descriptions

* Humans and animals were considered in the same scenario because human toxicology studies are derived from animal studies.

**Table 6:** Summary of the structured literature search for human toxicity.

Topic	Record
Records identified	428
Duplicate records removed	68
Title/Abstract Screening	360
Excluded	250
Peer-reviewed full-text articles	110
Excluded full-text	72 *
Papers older than 20 years	18
Final included studies	20

* Exclusion reasons reported in Table S3.

**Table 7:** Summary of the structured literature search on plant toxicity.

Topic	Record
Records identified	312
Duplicate records removed	52
Title/Abstract Screening	260
Excluded	180
Peer-reviewed full-text articles	80
Excluded full-text	48 *
Papers older than 20 years	9
Final included studies	23

* Exclusion reasons reported in Table S3.

**Table 8:** Summary of the structured literature search on environmental toxicity.

Topic	Record
Records identified	510
Duplicate records removed	90
Title/Abstract Screening	420
Excluded	290
Peer-reviewed full-text articles	130
Excluded full-text	98 *
Papers older than 20 years	15
Final included studies	17

* Exclusion reasons reported in Table S3.

## Data Availability

No new data were created or analyzed in this study. Data sharing is not applicable to this article.

## Supplementary Materials

The following supporting information can be downloaded at: https://www.mdpi.com/article/10.3390/toxics14070632/s1, Table S1: Main analytical methods used to estimate the bioavailable fraction of copper in soils; Table S2: Comparison of EU, US, Canadian/Australian, and international approaches to copper plant protection products; Table S3: Reasons for exclusion of full-text articles after eligibility assessment; Table S4: Level of evidence [150,151,152,153,154,155,156].
