# Supplementary material for: Copper Compounds: A Narrative and Regulatory Review of Agricultural Uses, Risks, and Environmental Assessment
Source: Toxics. 2026 Jul 20;14(7):632. doi: 10.3390/toxics14070632 (PMC13416924; doi:10.3390/toxics14070632)
Supplement: Supplementary file 1 [file toxics-14-00632-s001.zip › toxics-4355515-supplementary.pdf]

Table S1. Main analytical methods used to estimate the bioavailable fraction of copper in soils.

Method	Extractant / Principle	Copper fraction estimated	Main application	Advantages	Limitations
**Total digestion**	Aqua regia or HF digestion, and ICP-MS/OES analysis	Total copper content	Regulatory monitoring; contamination assessment	Standardized; measures total Cu concentration	Does not provide information on bioavailability
**$CaCl_2$ extraction**	0.01 M $CaCl_2$	Water-soluble and readily available copper	Estimation of immediately plant-available Cu	Simple, reproducible, and closely related to soil solution Cu	Extracts only a small fraction of total Cu; influenced by soil properties
**DTPA extraction**	0.005 M DTPA (pH 7.3)	Potentially plant-available copper	Agronomic assessment of Cu availability	Widely accepted for micronutrient evaluation; good correlation with plant uptake	May overestimate availability in some soils
**EDTA extraction**	EDTA chelating solution	Potentially mobilizable copper	Soil fertility and environmental assessment	Efficient extraction of exchangeable and weakly bound Cu	Less representative of the immediately bioavailable fraction
**NH_4NO_3 extraction**	1 M ammonium nitrate	Exchangeable copper	Environmental risk assessment	Standardized for mobile metals	Limited extraction efficiency for strongly sorbed Cu
**BCR sequential extraction**	Four-step sequential extraction	Exchangeable, reducible, oxidizable and residual fractions	Geochemical partitioning and mobility assessment	Provides information on copper speciation and potential mobility	Operationally defined fractions; not a direct measure of bioavailability
**DGT (Diffusive Gradients in Thin Films)**	Passive diffusion through a hydrogel	Labile copper available for resupply from soil	Environmental and ecotoxicological studies	Mimics plant uptake and correlates well with biological availability	More expensive and technically demanding
**Soil pore water analysis**	Extraction of soil solution followed by ICP-MS/OES	Dissolved copper species	Ecotoxicological and environmental studies	Measures the fraction directly available in soil solution	Sensitive to sampling and storage conditions; influenced by soil moisture

Abbreviations: DTPA, diethylenetriaminepentaacetic acid; EDTA, ethylenediaminetetraacetic acid; DGT, Diffusive Gradients in Thin Films; ICP-OES, Inductively Coupled Plasma Optical Emission Spectrometry; ICP-MS, Inductively Coupled Plasma Mass Spectrometry.

Table S2. Comparison of EU, US, Canadian/Australian, and international approaches to copper plant protection products.

Jurisdiction	PPP approval	Agricultural threshold	Dietary limits	Soil limits	Water quality criteria	Key references
**European Union**	Copper compounds approved under Regulation (EC) No 1107/2009; **Candidate for Substitution**; renewal subject to periodic EFSA review.	Maximum **28 kg Cu ha^{-1} over 7 years** (average 4 kg ha^{-1} $year^{-1}$); Member States may impose stricter restrictions.	MRLs established under Regulation (EC) No 396/2005; EFSA health-based guidance value (UL) **5 mg day^{-1}** for adults, ADI **0.07 mg Kg^{-1} bw day^{-1}**.	No harmonized EU agricultural soil limit; assessment based on accumulation, persistence, and ecological risk during PPP authorization.	Drinking water parametric value **2 mg L^{-1}**; aquatic risk assessed under PPP legislation and water legislation.	Regulation (EC) No 1107/2009 [94]; Regulation (EC) No 396/2005 [142]; Commission Implementing Regulation (EU) 2018/1981 [81]; EFSA (2022, 2023) [60, 62]; Directive (EU) 2020/2184 [147]; EFSA Peer review 2018 [15].
**United States**	Copper pesticides are registered and periodically reviewed by the EPA under FIFRA.	No national annual application limit; use is regulated by product labels and crop-specific maximum application rates.	EPA food tolerances; Institute of Medicine UL **10 mg day^{-1}** for adults.	No federal soil quality standard for agricultural copper; risk addressed during registration and state regulations.	EPA aquatic criteria based on the **Biotic Ligand Model (BLM)**; Lead and Copper Rule action level **1.3 mg L^{-1}** for drinking water.	EPA Copper facts [23]; EPA Registration Review of Copper Compounds [16]; EPA Lead and Copper Rule [150]; RED [151].
**Canada**	PMRA evaluates and registers copper pesticides under the Pest Control Products Act.	No national copper loading limit comparable to the EU.	Health Canada drinking water MAC **2 mg L^{-1}**; food MRLs established by PMRA.	Canadian Soil Quality Guidelines (CCME), land-use dependent.	Freshwater guidelines are hardness-dependent; drinking water MAC **2 mg L^{-1}**.	Health Canada (Guidelines for Canadian Drinking Water Quality) [152]; Copper and its compounds – information sheet [153]
**Australia / New Zealand**	APVMA (Australia) and ACVM/EPA (New Zealand) register copper pesticides.	No national annual copper application cap.	National MRLs for food; drinking water guideline **2 mg L^{-1}**.	Environmental guidelines managed by states and territories.	ANZG water-quality trigger values are bioavailability-dependent.	Australian and New Zealand guidelines for fresh and marine water quality [154].
**International (Codex/FAO/WHO)**	No global authorization system; Codex establishes international MRLs; FAO/WHO provides toxicological evaluations.	No international agricultural application limit.	WHO drinking water guideline **2 mg L^{-1}**;	No international harmonized soil limits.	WHO drinking water guideline **2 mg L^{-1}**.	Codex Alimentarius [155]; WHO Drinking-water Guidelines [156].

Table S3. Reasons for exclusion of full-text articles after eligibility assessment.

Method	Human	Soil	Plant	Reason
**Wrong population**	15	7	25	Animal or in vitro studies<br>No agricultural or natural soils<br>No higher plants or agricultural crops
**Wrong exposure**	7	5	10	Copper or copper-based plant protection products have not been evaluated, nor has exposure been quantified
**Wrong outcome**	8	8	15	No toxicological or health-related outcomes<br>No assessment of soil accumulation, mobility, bioavailability,<br>No assessment of phytotoxicity or copper accumulation
**Wrong study type**	20	8	20	Reviews, editorials, conference abstracts, book chapters
**Insufficient methodological information**	8	4	6	Experimental design or analytical methods are inadequately described
**No comparator / did not meet PECO criteria**	9	5	10	Studies did not satisfy the eligibility criteria
**Insufficient data**	5	11	12	The full text lacked usable data
**Total excluded**	72	48	98	

Table S4. Level of evidence.

Topic		Evidence*
Human	Neurotoxicity	High
	Nephrotoxicity	High
	Infertility	Moderate
	Hepatotoxicity	High
	Pro-oxidant	Moderate
	Cytotoxicity	Moderate
Microbial toxicity		Moderate
Fish toxicity		Moderate
Livestock toxicity		High
Soil accumulation		High
Plant toxicity		High
Long-term low-dose exposure		Low

* The level of evidence was defined considering the overall number of papers reporting each topic.
